# Di-μ-azido-κ^4^ *N* ^1^:*N* ^1^-bis­[(1,10-phen­anthroline-κ^2^ *N*,*N*′)(thio­cyanato-κ*N*)lead(II)]

**DOI:** 10.1107/S1600536810032125

**Published:** 2010-08-18

**Authors:** Gholamhossein Mohammadnezhad, Ali Reza Ghanbarpour, Mostafa M. Amini, Seik Weng Ng

**Affiliations:** a Department of Chemistry, General Campus, Shahid Beheshti University, Tehran 1983963113, Iran; b Department of Chemistry, University of Malaya, 50603 Kuala Lumpur, Malaysia

## Abstract

In the centrosymmetric binuclear title compound, [Pb_2_(N_3_)_2_(NCS)_2_(C_12_H_8_N_2_)_4_], the *N*-donor atoms of one *N*-heterocycle and the *N*-donor atom of a thio­cyanate anion along with the sterically active lone-pair electrons comprise an approximate square; a plane through three atoms of this square is twisted slightly with respect to the square made up of the other four atoms (two from the other *N*-heterocycle and one each from the bridging azide anions) at a dihedral angle of 18.7 (1)°. The Pb^II^ atom is in a Ψ-square-anti­prismaic coordination.

## Related literature

For related structures, see: Engelhardt *et al.* (1989 ▶); Zhu *et al.* (2008 ▶).
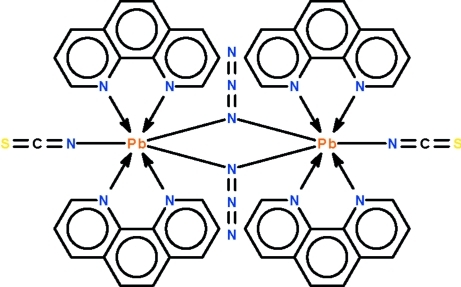


## Experimental

### 

#### Crystal data

[Pb_2_(N_3_)_2_(NCS)_2_(C_12_H_8_N_2_)_4_]
*M* *_r_* = 1335.42
Triclinic, 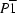

*a* = 10.3412 (6) Å
*b* = 10.8327 (6) Å
*c* = 11.4178 (6) Å
α = 89.923 (1)°
β = 72.080 (1)°
γ = 65.273 (1)°
*V* = 1093.35 (10) Å^3^
*Z* = 1
Mo *K*α radiation
μ = 7.85 mm^−1^
*T* = 100 K
0.25 × 0.15 × 0.15 mm

#### Data collection

Bruker SMART APEXII area-detector diffractometer
Absorption correction: multi-scan (*SADABS*; Sheldrick, 1996 ▶) *T*_min_ = 0.244, *T*_max_ = 0.386
10523 measured reflections
5002 independent reflections
4783 reflections with *I* > 2σ(*I*)
*R*_int_ = 0.021

#### Refinement

*R*[*F* ^2^ > 2σ(*F* ^2^)] = 0.020
*wR*(*F* ^2^) = 0.061
*S* = 1.09
5002 reflections
316 parameters
H-atom parameters constrained
Δρ_max_ = 1.23 e Å^−3^
Δρ_min_ = −1.38 e Å^−3^

### 

Data collection: *APEX2* (Bruker, 2009 ▶); cell refinement: *SAINT* (Bruker, 2009 ▶); data reduction: *SAINT*; program(s) used to solve structure: *SHELXS97* (Sheldrick, 2008 ▶); program(s) used to refine structure: *SHELXL97* (Sheldrick, 2008 ▶); molecular graphics: *X-SEED* (Barbour, 2001 ▶); software used to prepare material for publication: *publCIF* (Westrip, 2010 ▶).

## Supplementary Material

Crystal structure: contains datablocks global, I. DOI: 10.1107/S1600536810032125/ci5151sup1.cif

Structure factors: contains datablocks I. DOI: 10.1107/S1600536810032125/ci5151Isup2.hkl

Additional supplementary materials:  crystallographic information; 3D view; checkCIF report

## Figures and Tables

**Fig. 1.**
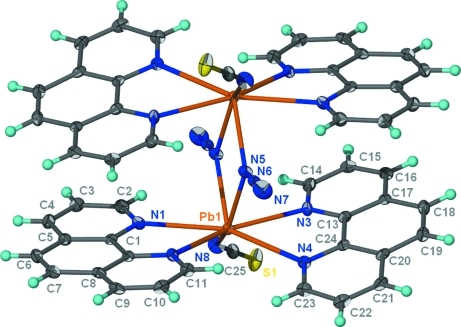
Displacement ellipsoid plot (Barbour, 2001) of centrosymmetric [Pb2(N3)2(NCS)2(C12H8N2)4] at the 70% probability level. H atoms are drawn as spheres of arbitrary radius. Symmetry-related (1 - x, 1 - y, 1 - z) atoms are not labeled.

**Fig. 2.**
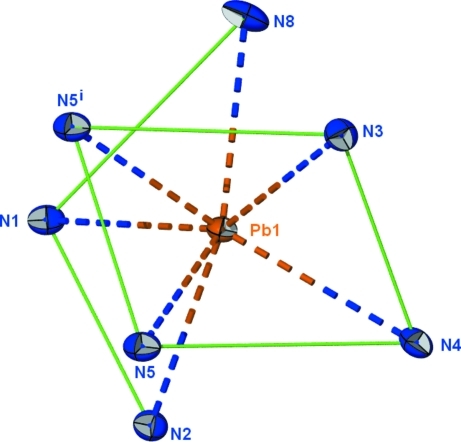
The square environment of the Pb atom.

## supplementary crystallographic information

### Comment

Relative to the (1,10-phenanthroline)lead(II) species, the azide counterion can function as a bridging unit through only one nitrogen atom only or through two end nitrogen atoms. In fact, an example is known in which the anion functions in both bridging modes (Zhu *et al.*, 2008). Similarly, an example is known in which the thiocyanate anion functions in dual bridging modes (Engelhardt *et al.*, 1989). The title compound (Scheme I) is a centrosymmetric dinuclear lead(II) compound having the azide and thiocyanate anions behaving only in one type of bonding interaction; the azide bridges through only one nitrogen atom but the thiocyanate anion is unidentate (Fig. 1). The nitrogen donor-atoms of one 1,10-phenanthroline ligand, the nitrogen donor-atom of a thiocyanate along with the sterically active lone-pair electrons comprise an approximate square (Fig. 2); the three atoms of this square is slightly twisted with respect to the square made up by the other four atoms (two from the other 1,10-phenanthroline and one each from the bridging azide anions) at a dihedral angle of 18.7 (1)°. Obviously, the tilt arises from the presence of the lone pair. The lead atom is displaced by 1.044 (3) Å with respect to the three-atom plane and is displaced in the opposite direction by 1.548 (1) Å with respect to the four atom plane. The geometry of the lead atom is better regarded as showing *Ψ*-square antiprismatic coordination.

### Experimental

Potassium thiocyanate (0.5 mmol, 0.09 g), sodium azide (0.03 g, 0.05 mmol) and 1,10-phenanthroline (2 mmol, 0.36 g) were loaded into one arm of a *U*-shaped glass tube. Lead(II) acetate (0.38 g, 1 mmol) and sodium nitrite (0.07 g, 1 mmol) were loaded into the other. Methanol was added to both arms. The ligand-containing arm was immersed in an oil bath at 333 K whereas the other arm was kept at ambient temperature. Crystals were collected after 10 days.

### Refinement

H atoms were placed in calculated positions (C–H = 0.95 Å) and included in the refinement in the riding model approximation, with *U_iso_*(H) set to 1.2 times *U*_eq_(C). The final difference Fourier map had a peak and a hole in the vicinity of the lead atom.

### Crystal data

**Table:**

[Pb_2_(N_3_)_2_(NCS)_2_(C_12_H_8_N_2_)_4_]	*Z* = 1
*M**_r_* = 1335.42	*F*(000) = 642
Triclinic, *P*1	*D*_x_ = 2.034 Mg m^−^^3^
Hall symbol: -P 1	Mo *K*α radiation, λ = 0.71073 Å
*a* = 10.3412 (6) Å	Cell parameters from 8054 reflections
*b* = 10.8327 (6) Å	θ = 2.3–28.3°
*c* = 11.4178 (6) Å	µ = 7.85 mm^−^^1^
α = 89.923 (1)°	*T* = 100 K
β = 72.080 (1)°	Prism, colourless
γ = 65.273 (1)°	0.25 × 0.15 × 0.15 mm
*V* = 1093.35 (10) Å^3^	

### Data collection

**Table:**

Bruker SMART APEXII area-detector diffractometer	5002 independent reflections
Radiation source: fine-focus sealed tube	4783 reflections with *I* > 2σ(*I*)
graphite	*R*_int_ = 0.021
ω scans	θ_max_ = 27.5°, θ_min_ = 1.9°
Absorption correction: multi-scan (*SADABS*; Sheldrick, 1996)	*h* = −13→13
*T*_min_ = 0.244, *T*_max_ = 0.386	*k* = −14→13
10523 measured reflections	*l* = −14→14

### Refinement

**Table:**

Refinement on *F*^2^	Primary atom site location: structure-invariant direct methods
Least-squares matrix: full	Secondary atom site location: difference Fourier map
*R*[*F*^2^ > 2σ(*F*^2^)] = 0.020	Hydrogen site location: inferred from neighbouring sites
*wR*(*F*^2^) = 0.061	H-atom parameters constrained
*S* = 1.09	*w* = 1/[σ^2^(*F*_o_^2^) + (0.041*P*)^2^] where *P* = (*F*_o_^2^ + 2*F*_c_^2^)/3
5002 reflections	(Δ/σ)_max_ = 0.001
316 parameters	Δρ_max_ = 1.23 e Å^−^^3^
0 restraints	Δρ_min_ = −1.37 e Å^−^^3^

### Fractional atomic coordinates and isotropic or equivalent isotropic displacement parameters (Å^2^)

**Table:**

	*x*	*y*	*z*	*U*_iso_*/*U*_eq_	
Pb1	0.451831 (12)	0.668463 (11)	0.618902 (10)	0.00916 (5)	
S1	−0.09428 (10)	0.84240 (11)	0.95648 (9)	0.0222 (2)	
N1	0.3623 (3)	0.8343 (3)	0.4496 (3)	0.0121 (6)	
N2	0.6474 (3)	0.7683 (3)	0.4566 (3)	0.0114 (6)	
N3	0.4103 (3)	0.4959 (3)	0.7794 (3)	0.0120 (6)	
N4	0.6519 (3)	0.5550 (3)	0.7575 (3)	0.0120 (6)	
N5	0.6529 (3)	0.4519 (3)	0.4832 (3)	0.0123 (6)	
N6	0.7855 (3)	0.4215 (3)	0.4627 (3)	0.0126 (6)	
N7	0.9113 (4)	0.3913 (3)	0.4423 (3)	0.0233 (7)	
N8	0.1509 (3)	0.8079 (3)	0.7442 (3)	0.0192 (6)	
C1	0.4430 (4)	0.8968 (3)	0.3809 (3)	0.0105 (6)	
C2	0.2277 (4)	0.8640 (4)	0.4419 (4)	0.0155 (7)	
H2	0.1709	0.8205	0.4904	0.019*	
C3	0.1637 (4)	0.9559 (4)	0.3663 (3)	0.0142 (7)	
H3	0.0672	0.9724	0.3631	0.017*	
C4	0.2422 (4)	1.0207 (4)	0.2979 (3)	0.0151 (7)	
H4	0.2006	1.0841	0.2468	0.018*	
C5	0.3852 (4)	0.9931 (4)	0.3035 (3)	0.0124 (6)	
C6	0.4768 (4)	1.0539 (3)	0.2299 (3)	0.0137 (7)	
H6	0.4386	1.1174	0.1775	0.016*	
C7	0.6157 (4)	1.0224 (4)	0.2339 (3)	0.0135 (7)	
H7	0.6738	1.0641	0.1844	0.016*	
C8	0.6776 (4)	0.9273 (3)	0.3113 (3)	0.0120 (6)	
C9	0.8244 (4)	0.8902 (4)	0.3146 (3)	0.0141 (7)	
H9	0.8849	0.9309	0.2670	0.017*	
C10	0.8783 (4)	0.7946 (4)	0.3876 (3)	0.0151 (7)	
H10	0.9766	0.7682	0.3915	0.018*	
C11	0.7866 (4)	0.7369 (4)	0.4559 (3)	0.0138 (7)	
H11	0.8263	0.6703	0.5054	0.017*	
C12	0.5929 (4)	0.8631 (3)	0.3841 (3)	0.0098 (6)	
C13	0.5152 (4)	0.4199 (4)	0.8317 (3)	0.0115 (6)	
C14	0.2921 (4)	0.4702 (4)	0.7950 (3)	0.0141 (7)	
H14	0.2183	0.5243	0.7597	0.017*	
C15	0.2702 (4)	0.3671 (4)	0.8612 (3)	0.0133 (7)	
H15	0.1833	0.3527	0.8706	0.016*	
C16	0.3759 (4)	0.2880 (4)	0.9118 (3)	0.0136 (7)	
H16	0.3639	0.2172	0.9563	0.016*	
C17	0.5034 (4)	0.3128 (3)	0.8971 (3)	0.0113 (6)	
C18	0.6175 (4)	0.2330 (4)	0.9489 (3)	0.0145 (7)	
H18	0.6107	0.1584	0.9898	0.017*	
C19	0.7349 (4)	0.2629 (4)	0.9401 (3)	0.0142 (7)	
H19	0.8093	0.2095	0.9752	0.017*	
C20	0.7469 (4)	0.3743 (4)	0.8785 (3)	0.0125 (7)	
C21	0.8638 (4)	0.4121 (4)	0.8715 (3)	0.0145 (7)	
H21	0.9364	0.3640	0.9100	0.017*	
C22	0.8727 (4)	0.5168 (4)	0.8102 (3)	0.0150 (7)	
H22	0.9505	0.5435	0.8057	0.018*	
C23	0.7647 (4)	0.5852 (4)	0.7531 (3)	0.0139 (7)	
H23	0.7732	0.6573	0.7089	0.017*	
C24	0.6409 (4)	0.4512 (3)	0.8211 (3)	0.0102 (6)	
C25	0.0496 (4)	0.8222 (4)	0.8327 (3)	0.0142 (7)	

### Atomic displacement parameters (Å^2^)

**Table:**

	*U*^11^	*U*^22^	*U*^33^	*U*^12^	*U*^13^	*U*^23^
Pb1	0.00832 (7)	0.01057 (7)	0.00924 (8)	−0.00546 (5)	−0.00197 (5)	0.00214 (5)
S1	0.0135 (4)	0.0343 (5)	0.0161 (5)	−0.0093 (4)	−0.0034 (4)	0.0113 (4)
N1	0.0106 (13)	0.0119 (14)	0.0143 (14)	−0.0061 (11)	−0.0032 (11)	0.0025 (11)
N2	0.0114 (13)	0.0113 (14)	0.0117 (14)	−0.0057 (11)	−0.0030 (11)	0.0027 (11)
N3	0.0125 (13)	0.0134 (14)	0.0113 (14)	−0.0069 (11)	−0.0040 (11)	0.0053 (11)
N4	0.0122 (13)	0.0137 (14)	0.0096 (14)	−0.0071 (11)	−0.0010 (11)	0.0016 (11)
N5	0.0106 (13)	0.0121 (14)	0.0144 (14)	−0.0064 (11)	−0.0023 (11)	0.0008 (11)
N6	0.0145 (14)	0.0113 (14)	0.0143 (15)	−0.0075 (11)	−0.0055 (12)	0.0026 (11)
N7	0.0137 (15)	0.0235 (17)	0.034 (2)	−0.0092 (13)	−0.0084 (14)	0.0047 (15)
N8	0.0147 (15)	0.0193 (16)	0.0184 (16)	−0.0066 (13)	−0.0002 (13)	0.0012 (13)
C1	0.0117 (15)	0.0119 (16)	0.0044 (14)	−0.0051 (13)	0.0016 (12)	−0.0017 (12)
C2	0.0154 (16)	0.0155 (18)	0.0184 (18)	−0.0087 (14)	−0.0069 (14)	0.0040 (14)
C3	0.0108 (15)	0.0165 (17)	0.0145 (17)	−0.0050 (14)	−0.0047 (13)	0.0010 (14)
C4	0.0159 (17)	0.0148 (17)	0.0142 (17)	−0.0049 (14)	−0.0073 (14)	0.0030 (14)
C5	0.0131 (15)	0.0112 (16)	0.0104 (16)	−0.0046 (13)	−0.0019 (13)	−0.0002 (13)
C6	0.0158 (16)	0.0100 (16)	0.0118 (16)	−0.0036 (13)	−0.0029 (13)	0.0023 (13)
C7	0.0152 (16)	0.0137 (16)	0.0091 (16)	−0.0086 (13)	0.0021 (13)	0.0001 (13)
C8	0.0121 (15)	0.0098 (16)	0.0107 (16)	−0.0045 (13)	0.0002 (13)	−0.0021 (13)
C9	0.0134 (16)	0.0115 (16)	0.0160 (17)	−0.0075 (13)	−0.0003 (13)	−0.0009 (13)
C10	0.0109 (15)	0.0179 (18)	0.0142 (17)	−0.0048 (14)	−0.0034 (13)	−0.0014 (14)
C11	0.0120 (15)	0.0138 (17)	0.0136 (17)	−0.0052 (13)	−0.0025 (13)	0.0008 (13)
C12	0.0103 (15)	0.0103 (15)	0.0057 (15)	−0.0043 (12)	0.0012 (12)	−0.0007 (12)
C13	0.0110 (15)	0.0127 (16)	0.0093 (16)	−0.0042 (13)	−0.0028 (12)	−0.0004 (13)
C14	0.0138 (16)	0.0175 (17)	0.0138 (17)	−0.0080 (14)	−0.0068 (13)	0.0038 (14)
C15	0.0120 (15)	0.0181 (17)	0.0114 (16)	−0.0108 (14)	0.0001 (13)	−0.0003 (13)
C16	0.0159 (16)	0.0131 (16)	0.0075 (16)	−0.0069 (14)	0.0023 (13)	−0.0009 (13)
C17	0.0136 (15)	0.0105 (16)	0.0053 (15)	−0.0039 (13)	0.0006 (12)	−0.0022 (12)
C18	0.0161 (16)	0.0130 (17)	0.0112 (16)	−0.0047 (14)	−0.0031 (13)	0.0037 (13)
C19	0.0140 (16)	0.0143 (17)	0.0125 (16)	−0.0043 (13)	−0.0047 (13)	0.0027 (13)
C20	0.0123 (16)	0.0148 (17)	0.0069 (15)	−0.0040 (13)	−0.0016 (13)	−0.0025 (13)
C21	0.0120 (15)	0.0172 (17)	0.0122 (16)	−0.0039 (13)	−0.0050 (13)	−0.0004 (13)
C22	0.0100 (15)	0.0227 (19)	0.0120 (16)	−0.0094 (14)	0.0000 (13)	−0.0008 (14)
C23	0.0118 (15)	0.0165 (17)	0.0115 (16)	−0.0077 (13)	0.0004 (13)	0.0006 (13)
C24	0.0100 (15)	0.0120 (16)	0.0061 (15)	−0.0043 (13)	−0.0003 (12)	−0.0009 (12)
C25	0.0127 (16)	0.0134 (17)	0.0163 (17)	−0.0048 (13)	−0.0059 (14)	0.0043 (13)

### Geometric parameters (Å, °)

**Table:**

Pb1—N5	2.485 (3)	C6—H6	0.95
Pb1—N5^i^	2.489 (3)	C7—C8	1.430 (5)
Pb1—N3	2.687 (3)	C7—H7	0.95
Pb1—N8	2.711 (3)	C8—C12	1.415 (5)
Pb1—N1	2.742 (3)	C8—C9	1.412 (5)
Pb1—N4	2.860 (3)	C9—C10	1.370 (5)
Pb1—N2	2.865 (3)	C9—H9	0.95
S1—C25	1.641 (4)	C10—C11	1.392 (5)
N1—C2	1.324 (4)	C10—H10	0.95
N1—C1	1.356 (4)	C11—H11	0.95
N2—C11	1.331 (4)	C13—C17	1.407 (5)
N2—C12	1.364 (4)	C13—C24	1.446 (5)
N3—C14	1.324 (4)	C14—C15	1.404 (5)
N3—C13	1.364 (4)	C14—H14	0.95
N4—C23	1.325 (4)	C15—C16	1.366 (5)
N4—C24	1.363 (4)	C15—H15	0.95
N5—N6	1.211 (4)	C16—C17	1.413 (5)
N5—Pb1^i^	2.489 (3)	C16—H16	0.95
N6—N7	1.145 (4)	C17—C18	1.437 (5)
N8—C25	1.164 (5)	C18—C19	1.358 (5)
C1—C5	1.423 (5)	C18—H18	0.95
C1—C12	1.448 (5)	C19—C20	1.427 (5)
C2—C3	1.404 (5)	C19—H19	0.95
C2—H2	0.95	C20—C21	1.412 (5)
C3—C4	1.359 (5)	C20—C24	1.415 (5)
C3—H3	0.95	C21—C22	1.353 (5)
C4—C5	1.403 (5)	C21—H21	0.95
C4—H4	0.95	C22—C23	1.405 (5)
C5—C6	1.439 (5)	C22—H22	0.95
C6—C7	1.347 (5)	C23—H23	0.95
			
N5—Pb1—N5^i^	67.99 (11)	C6—C7—C8	121.3 (3)
N5—Pb1—N3	82.92 (9)	C6—C7—H7	119.3
N5^i^—Pb1—N3	77.99 (9)	C8—C7—H7	119.3
N5—Pb1—N8	144.35 (10)	C12—C8—C9	118.0 (3)
N5^i^—Pb1—N8	78.18 (10)	C12—C8—C7	119.8 (3)
N3—Pb1—N8	79.30 (9)	C9—C8—C7	122.2 (3)
N5—Pb1—N1	102.42 (9)	C10—C9—C8	118.9 (3)
N5^i^—Pb1—N1	76.63 (9)	C10—C9—H9	120.5
N3—Pb1—N1	149.70 (8)	C8—C9—H9	120.5
N8—Pb1—N1	79.54 (9)	C9—C10—C11	119.0 (3)
N5—Pb1—N4	76.52 (9)	C9—C10—H10	120.5
N5^i^—Pb1—N4	127.42 (9)	C11—C10—H10	120.5
N3—Pb1—N4	59.80 (8)	N2—C11—C10	124.6 (3)
N8—Pb1—N4	118.75 (9)	N2—C11—H11	117.7
N1—Pb1—N4	150.49 (8)	C10—C11—H11	117.7
N5—Pb1—N2	79.06 (9)	N2—C12—C8	122.6 (3)
N5^i^—Pb1—N2	116.13 (9)	N2—C12—C1	118.0 (3)
N3—Pb1—N2	150.03 (8)	C8—C12—C1	119.4 (3)
N8—Pb1—N2	127.96 (9)	N3—C13—C17	121.7 (3)
N1—Pb1—N2	58.79 (8)	N3—C13—C24	119.2 (3)
N4—Pb1—N2	92.59 (8)	C17—C13—C24	119.0 (3)
C2—N1—C1	118.1 (3)	N3—C14—C15	123.3 (3)
C2—N1—Pb1	118.2 (2)	N3—C14—H14	118.3
C1—N1—Pb1	123.5 (2)	C15—C14—H14	118.3
C11—N2—C12	116.9 (3)	C16—C15—C14	118.9 (3)
C11—N2—Pb1	123.4 (2)	C16—C15—H15	120.5
C12—N2—Pb1	119.4 (2)	C14—C15—H15	120.5
C14—N3—C13	118.5 (3)	C15—C16—C17	119.2 (3)
C14—N3—Pb1	117.9 (2)	C15—C16—H16	120.4
C13—N3—Pb1	123.0 (2)	C17—C16—H16	120.4
C23—N4—C24	117.7 (3)	C16—C17—C13	118.3 (3)
C23—N4—Pb1	124.6 (2)	C16—C17—C18	121.4 (3)
C24—N4—Pb1	117.2 (2)	C13—C17—C18	120.3 (3)
N6—N5—Pb1	121.1 (2)	C19—C18—C17	120.8 (3)
N6—N5—Pb1^i^	126.5 (2)	C19—C18—H18	119.6
Pb1—N5—Pb1^i^	112.01 (11)	C17—C18—H18	119.6
N7—N6—N5	179.1 (4)	C18—C19—C20	120.2 (3)
C25—N8—Pb1	147.1 (3)	C18—C19—H19	119.9
N1—C1—C5	121.6 (3)	C20—C19—H19	119.9
N1—C1—C12	119.4 (3)	C21—C20—C24	117.3 (3)
C5—C1—C12	119.0 (3)	C21—C20—C19	122.0 (3)
N1—C2—C3	123.8 (3)	C24—C20—C19	120.7 (3)
N1—C2—H2	118.1	C22—C21—C20	120.1 (3)
C3—C2—H2	118.1	C22—C21—H21	120.0
C4—C3—C2	118.9 (3)	C20—C21—H21	120.0
C4—C3—H3	120.5	C21—C22—C23	118.7 (3)
C2—C3—H3	120.5	C21—C22—H22	120.6
C3—C4—C5	119.3 (3)	C23—C22—H22	120.6
C3—C4—H4	120.4	N4—C23—C22	123.8 (3)
C5—C4—H4	120.4	N4—C23—H23	118.1
C4—C5—C1	118.3 (3)	C22—C23—H23	118.1
C4—C5—C6	122.2 (3)	N4—C24—C20	122.4 (3)
C1—C5—C6	119.5 (3)	N4—C24—C13	118.8 (3)
C7—C6—C5	121.1 (3)	C20—C24—C13	118.8 (3)
C7—C6—H6	119.5	N8—C25—S1	179.4 (3)
C5—C6—H6	119.5		
			
N5—Pb1—N1—C2	109.1 (3)	C1—N1—C2—C3	0.3 (5)
N5^i^—Pb1—N1—C2	45.6 (3)	Pb1—N1—C2—C3	174.9 (3)
N3—Pb1—N1—C2	11.8 (4)	N1—C2—C3—C4	−1.1 (6)
N8—Pb1—N1—C2	−34.6 (3)	C2—C3—C4—C5	0.7 (5)
N4—Pb1—N1—C2	−166.4 (2)	C3—C4—C5—C1	0.3 (5)
N2—Pb1—N1—C2	178.0 (3)	C3—C4—C5—C6	177.5 (3)
N5—Pb1—N1—C1	−76.6 (3)	N1—C1—C5—C4	−1.1 (5)
N5^i^—Pb1—N1—C1	−140.1 (3)	C12—C1—C5—C4	177.6 (3)
N3—Pb1—N1—C1	−173.9 (2)	N1—C1—C5—C6	−178.4 (3)
N8—Pb1—N1—C1	139.7 (3)	C12—C1—C5—C6	0.4 (5)
N4—Pb1—N1—C1	7.9 (4)	C4—C5—C6—C7	−177.9 (3)
N2—Pb1—N1—C1	−7.8 (2)	C1—C5—C6—C7	−0.7 (5)
N5—Pb1—N2—C11	−67.0 (3)	C5—C6—C7—C8	0.0 (5)
N5^i^—Pb1—N2—C11	−125.6 (3)	C6—C7—C8—C12	0.9 (5)
N3—Pb1—N2—C11	−12.9 (4)	C6—C7—C8—C9	178.4 (3)
N8—Pb1—N2—C11	139.0 (3)	C12—C8—C9—C10	−0.1 (5)
N1—Pb1—N2—C11	−178.9 (3)	C7—C8—C9—C10	−177.6 (3)
N4—Pb1—N2—C11	8.8 (3)	C8—C9—C10—C11	0.2 (5)
N5—Pb1—N2—C12	119.5 (2)	C12—N2—C11—C10	0.6 (5)
N5^i^—Pb1—N2—C12	60.9 (3)	Pb1—N2—C11—C10	−173.1 (3)
N3—Pb1—N2—C12	173.6 (2)	C9—C10—C11—N2	−0.4 (6)
N8—Pb1—N2—C12	−34.5 (3)	C11—N2—C12—C8	−0.5 (5)
N1—Pb1—N2—C12	7.6 (2)	Pb1—N2—C12—C8	173.4 (2)
N4—Pb1—N2—C12	−164.7 (2)	C11—N2—C12—C1	178.6 (3)
N5—Pb1—N3—C14	−104.1 (3)	Pb1—N2—C12—C1	−7.5 (4)
N5^i^—Pb1—N3—C14	−35.1 (3)	C9—C8—C12—N2	0.3 (5)
N8—Pb1—N3—C14	44.9 (3)	C7—C8—C12—N2	177.8 (3)
N1—Pb1—N3—C14	−1.5 (4)	C9—C8—C12—C1	−178.8 (3)
N4—Pb1—N3—C14	177.4 (3)	C7—C8—C12—C1	−1.2 (5)
N2—Pb1—N3—C14	−157.3 (2)	N1—C1—C12—N2	0.3 (5)
N5—Pb1—N3—C13	66.6 (3)	C5—C1—C12—N2	−178.5 (3)
N5^i^—Pb1—N3—C13	135.5 (3)	N1—C1—C12—C8	179.4 (3)
N8—Pb1—N3—C13	−144.4 (3)	C5—C1—C12—C8	0.6 (5)
N1—Pb1—N3—C13	169.2 (2)	C14—N3—C13—C17	2.3 (5)
N4—Pb1—N3—C13	−11.9 (2)	Pb1—N3—C13—C17	−168.3 (2)
N2—Pb1—N3—C13	13.4 (4)	C14—N3—C13—C24	−176.8 (3)
N5—Pb1—N4—C23	93.2 (3)	Pb1—N3—C13—C24	12.6 (4)
N5^i^—Pb1—N4—C23	141.3 (3)	C13—N3—C14—C15	−1.0 (5)
N3—Pb1—N4—C23	−177.2 (3)	Pb1—N3—C14—C15	170.1 (3)
N8—Pb1—N4—C23	−121.5 (3)	N3—C14—C15—C16	−0.4 (6)
N1—Pb1—N4—C23	1.8 (4)	C14—C15—C16—C17	0.5 (5)
N2—Pb1—N4—C23	15.1 (3)	C15—C16—C17—C13	0.8 (5)
N5—Pb1—N4—C24	−78.8 (2)	C15—C16—C17—C18	179.7 (3)
N5^i^—Pb1—N4—C24	−30.8 (3)	N3—C13—C17—C16	−2.2 (5)
N3—Pb1—N4—C24	10.8 (2)	C24—C13—C17—C16	176.9 (3)
N8—Pb1—N4—C24	66.4 (2)	N3—C13—C17—C18	178.8 (3)
N1—Pb1—N4—C24	−170.3 (2)	C24—C13—C17—C18	−2.1 (5)
N2—Pb1—N4—C24	−156.9 (2)	C16—C17—C18—C19	−176.2 (3)
N5^i^—Pb1—N5—N6	173.0 (3)	C13—C17—C18—C19	2.7 (5)
N3—Pb1—N5—N6	−107.1 (3)	C17—C18—C19—C20	−0.3 (5)
N8—Pb1—N5—N6	−167.5 (2)	C18—C19—C20—C21	177.5 (3)
N1—Pb1—N5—N6	103.1 (3)	C18—C19—C20—C24	−2.7 (5)
N4—Pb1—N5—N6	−46.6 (3)	C24—C20—C21—C22	−1.0 (5)
N2—Pb1—N5—N6	48.8 (3)	C19—C20—C21—C22	178.8 (3)
N5^i^—Pb1—N5—Pb1^i^	0.0	C20—C21—C22—C23	−0.6 (5)
N3—Pb1—N5—Pb1^i^	79.87 (12)	C24—N4—C23—C22	−0.1 (5)
N8—Pb1—N5—Pb1^i^	19.5 (2)	Pb1—N4—C23—C22	−172.1 (3)
N1—Pb1—N5—Pb1^i^	−69.85 (12)	C21—C22—C23—N4	1.2 (5)
N4—Pb1—N5—Pb1^i^	140.43 (13)	C23—N4—C24—C20	−1.6 (5)
N2—Pb1—N5—Pb1^i^	−124.19 (12)	Pb1—N4—C24—C20	171.0 (2)
N5—Pb1—N8—C25	79.2 (6)	C23—N4—C24—C13	177.5 (3)
N5^i^—Pb1—N8—C25	97.7 (5)	Pb1—N4—C24—C13	−9.9 (4)
N3—Pb1—N8—C25	17.8 (5)	C21—C20—C24—N4	2.2 (5)
N1—Pb1—N8—C25	176.0 (5)	C19—C20—C24—N4	−177.6 (3)
N4—Pb1—N8—C25	−28.7 (6)	C21—C20—C24—C13	−176.9 (3)
N2—Pb1—N8—C25	−148.3 (5)	C19—C20—C24—C13	3.3 (5)
C2—N1—C1—C5	0.8 (5)	N3—C13—C24—N4	−0.9 (5)
Pb1—N1—C1—C5	−173.5 (2)	C17—C13—C24—N4	180.0 (3)
C2—N1—C1—C12	−177.9 (3)	N3—C13—C24—C20	178.3 (3)
Pb1—N1—C1—C12	7.8 (4)	C17—C13—C24—C20	−0.9 (5)

Symmetry codes: (i) −*x*+1, −*y*+1, −*z*+1.
